# Mental health of people living in Afghanistan: protocol for a systematic review

**DOI:** 10.1136/bmjopen-2026-122310

**Published:** 2026-07-31

**Authors:** Heela Saeed, Batu Kaya, Isla Kuhn, Faraz Mughal, Mohammad Sharif Razai

**Affiliations:** 1 Ibn-Sina Medical University, Kabul, Afghanistan; 2 Massey College, University of Toronto, Toronto, Ontario, Canada; 3 School of Clinical Medicine, University of Cambridge, Cambridge, UK; 4 Oxford Primary Care Clinical Trials Unit, Nuffield Department of Primary Care Health Sciences, University of Oxford, Oxford, UK; 5 Primary Care Unit, Department of Public Health and Primary Care, University of Cambridge, Cambridge, Cambridgeshire, UK

**Keywords:** MENTAL HEALTH, Coping Strategies, Psychosocial Intervention

## Abstract

**Abstract:**

**Introduction:**

Afghanistan has experienced decades of war, political instability, displacement, poverty and structural upheaval, all of which are likely to have contributed to the population’s deteriorating mental health. Individual studies and reports describe depression, anxiety, trauma-related distress and a severe lack of access to care, especially among women. However, the evidence remains fragmented. There has been no recent synthesis of evidence on mental health conditions, determinants, coping strategies, services and interventions among people living in Afghanistan. This protocol describes a systematic review of the literature on the mental health of this population.

**Methods and analysis:**

This systematic review protocol follows the Preferred Reporting Items for Systematic Review and Meta-Analysis Protocols (PRISMA-P) checklist. The review will be conducted and reported in accordance with PRISMA 2020. MEDLINE, Embase, APA PsycINFO, Web of Science and WHO Global Index Medicus will be searched using subject headings and keywords related to Afghanistan and mental health, with searches updated before final analysis. Relevant grey literature will also be included. Eligible study designs include quantitative, qualitative and mixed-methods studies reporting mental health outcomes, determinants, coping strategies, access to mental healthcare or mental health interventions among people living in Afghanistan. No language restrictions will be applied. Title and abstract screening and full-text review will be conducted independently by two reviewers, with disagreements resolved through discussion or, if necessary, adjudicated by a third reviewer. Data will be extracted using a piloted structured template. Given the expected heterogeneity in study designs and outcome measures, quantitative findings will be reported as frequencies and qualitative data will be analysed using thematic approaches to support the synthesis. Where appropriate, findings will be grouped by population subgroup and study setting. Study quality will be appraised using the Mixed Methods Appraisal Tool.

**Ethics and dissemination:**

Ethical approval is not required for this study as it will involve the analysis of previously published literature and publicly available reports. The findings will be disseminated through peer-reviewed publication and conference presentations and may inform clinicians, researchers, humanitarian agencies and policymakers.

**PROSPERO registration number:**

CRD420261403755.

**STRENGTHS AND LIMITATIONS OF THIS STUDY**

- The protocol follows Preferred Reporting Items for Systematic Review and Meta-Analysis Protocols (PRISMA-P), and the review will be reported in accordance with PRISMA 2020.
- Searches will cover bibliographic databases, reference lists and grey literature with no language restrictions.
- The systematic review will include quantitative, qualitative and mixed-methods studies using predefined Sample, Phenomenon of Interest, Design, Evaluation, Research type (SPIDER) eligibility criteria.
- Heterogeneity in study design, populations, measures and settings is likely to limit comparability and may preclude meta-analysis.

## Introduction

 Afghanistan has experienced decades of conflict, political instability and poverty, exposing its population to violence, injury and forced displacement. High levels of trauma exposure have been reported, with an estimated 86% of individuals experiencing or witnessing at least one traumatic event.^1^ Psychological distress is common, with women and children disproportionately affected.^2^

The return of the Taliban to power in August 2021 precipitated widespread changes in political, economic and social conditions. Rights and freedoms have been restricted, particularly for women and girls, including limitations on education, employment and mobility.^3 4^ At the same time, reductions in international aid and the departure of healthcare professionals have further weakened health and social systems.^4 5^

These structural restrictions have caused worsening mental health risks.^6–8^ For example, a large cross-sectional study among 2698 people across three regions of Afghanistan used the 21-item Depression, Anxiety and Stress Scale and found that 72% of participants had symptoms of depression, 71.9% had symptoms of anxiety and 66% experienced stress.^7^ Another study among Afghan women found that 80% of 438 participants experienced symptoms of depression and anxiety.^6^ Self-harm and suicidal behaviours are also serious concerns, though they remain underestimated in Afghanistan.^9^ However, these studies were based on geographically or demographically limited samples, so their findings may not be nationally representative.

A 2023 thematic review examined four decades of research and interventions on Afghan mental health and psychosocial well-being, with 214 included studies from 1978 to July 2022.^8^ The review found higher mental health risks among women, ethnic minorities, people with disabilities and youth; described culturally specific idioms of distress linking body and mind; and identified faith and family as central coping resources. It also documented efforts to integrate mental health into healthcare, train psychosocial counsellors and develop community-based psychosocial initiatives, although intervention evidence remained limited.^8^ Importantly, the number of psychiatrists in Afghanistan increased from just 2 in 2002 to over 100 in 2019. However, the mental health system remained far below acceptable levels of service provision.^8^

Many Afghans are reluctant to disclose mental health problems due to stigma, cultural and religious beliefs, shame and fear of punishment. Some seek help from psychiatrists or traditional healers, while others turn to religious practices and family support. Women report a range of coping strategies, such as drawing, painting, writing poetry, participating in small family gatherings, religious practice and meditation.^10^

Although individual studies and reports describe mental health conditions, determinants, coping strategies and service provision, the evidence is fragmented across disciplines and sources. A focused synthesis is needed to bring together current evidence on mental health among people living in Afghanistan after the political, social, economic and health-system changes that followed August 2021. This period marked a major change in governance, increasing restrictions on education, employment and mobility for women, reductions in international aid and disruption to health and social systems. A four-decade thematic review has already synthesised earlier Afghan mental health research and interventions, including literature identified up to July 2022.^8^

### Objective

This protocol describes a systematic review of studies on the mental health of people living in Afghanistan. The review aims to synthesise evidence on reported mental health conditions, social and structural determinants, coping strategies, access to mental healthcare and interventions intended to improve mental health outcomes, and to identify gaps in the evidence base. A focused synthesis is needed to compare findings and clarify what is known and what remains uncertain in light of recent events. Questions on prevalence, patterns and subgroup differences are primarily descriptive, while questions on associated factors, coping strategies, help-seeking pathways and intervention evidence are primarily explanatory.

## Methods

### Reporting guidelines

The systematic review will be conducted and reported in accordance with the Preferred Reporting Items for Systematic Review and Meta-Analysis (PRISMA) 2020.^11^ This review protocol follows the PRISMA Protocols (PRISMA-P) checklist (online supplemental file 1). The protocol is registered with the International Prospective Register of Systematic Reviews (PROSPERO) CRD420261403755.

### Research questions

The review will address the following questions:

1. What are the reported prevalence, levels and patterns of mental health problems among people living in Afghanistan?
2. What social, economic, political and health system factors are associated with mental health outcomes in this population?
3. What coping strategies, sources of support and help-seeking pathways are reported by this population?
4. What evidence exists regarding mental health services and interventions in Afghanistan?
5. How do findings vary by sex, ethnicity and population subgroup (eg, age, geographical setting, religious and linguistic minorities) where reported?

### Eligibility criteria

We will include studies reporting on the mental health of people living in Afghanistan. The review questions and search strategy were developed with support from an information specialist (IK) using the SPIDER framework: Sample, Phenomenon of Interest, Design, Evaluation and Research type (table 1).^12^ This framework was chosen because the review will include qualitative, quantitative and mixed-methods evidence.

**Table 1:** Studies eligible for inclusion will meet the following criteria

SPIDER element	Description
Sample	People of any age living in Afghanistan
Phenomenon of Interest	Mental health conditions, psychological distress, psychosocial well-being, determinants of mental health, coping strategies, help-seeking, access to mental healthcare, mental health services or mental health interventions
Design	Qualitative studies, quantitative observational studies, interventional studies and mixed-methods studies
Evaluation	Reported mental health outcomes; lived experiences of mental ill-health or well-being; determinants or correlates of mental health; service access, utilisation, acceptability or perceived effectiveness; intervention outcomes
Research type	Qualitative, quantitative and mixed methods

Studies published from 2021 onwards will be included. No language restrictions will be applied. Non-English articles will be screened using translation by reviewers with relevant language expertise, or professional translation if feasible. Machine translation may be used where appropriate.

Studies will be excluded if they:

- Focus only on Afghan refugees or other Afghan populations outside Afghanistan.
- Focus only on non-Afghan groups, such as veterans.
- Include Afghans within a wider sample but do not report data separately for those living in Afghanistan.
- Are opinion pieces, commentaries, case reports, editorials or narrative essays without original empirical data.
- Do not report mental health, psychosocial well-being, determinants, coping, service use or intervention findings relevant to the review questions.

### Information sources

**Figure 1:**
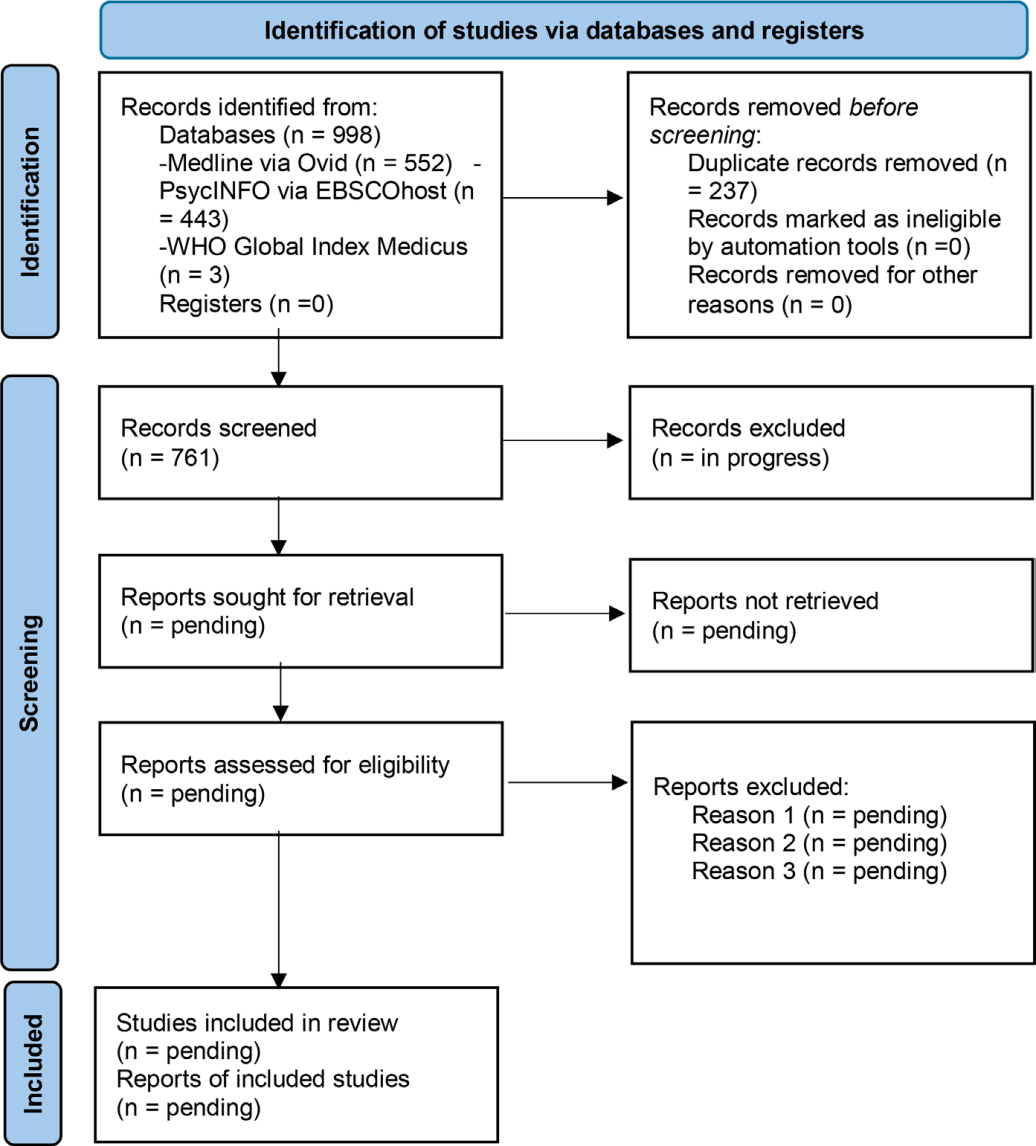
Preliminary preferred reporting items for systematic reviews and meta-analyses (PRISMA) flow diagram.

A comprehensive search will be conducted in MEDLINE, Embase, APA PsycINFO, Web of Science and WHO Global Index Medicus. Searches will be updated before final synthesis. Grey literature will be searched using Google Scholar and relevant institutional websites, including United Nations, WHO and humanitarian agency websites. Figure 1 provides the preliminary PRISMA flow diagram summarising the study selection process. Reference lists of included studies and relevant review articles will be screened manually to identify additional eligible records.

### Search strategy

The search strategy was developed and refined with support from an information specialist (IK). The full search strategy for MEDLINE, APA PsycINFO and WHO Global Index Medicus is provided in online supplemental file 2. Search terms combined subject headings and free-text terms related to Afghanistan and mental health. Terms for specific conditions and constructs, including depression, anxiety, stress, post-traumatic stress, trauma, psychosocial well-being, distress, coping, help-seeking, mental health services, self-harm, self-injurious behaviour and suicide, were incorporated as appropriate. Search strategies will be adapted for each database.

### Data selection and extraction

All identified records will be imported into a reference management system, and duplicate entries will be removed. Records will then be uploaded to Rayyan for screening.^13^ Titles and abstracts will be screened independently by two reviewers against the eligibility criteria. Full-texts of sources deemed potentially relevant will then be reviewed. Screening will be conducted independently by two reviewers to minimise selection bias. Any disagreements will be resolved through discussion. If consensus cannot be reached, a third reviewer will adjudicate.^14^ Reasons for exclusion at full-text stage will be recorded and reported in the PRISMA flow diagram.

Data extraction will be guided by a standardised data extraction form developed for this review and will be piloted. Extracted information will include publication characteristics (author, year and source type), study design, population characteristics, reported mental health outcomes, identified social, economic and political determinants, documented coping or adaptive strategies, descriptions of available mental health services or interventions, and key recommendations. For qualitative studies, extracted data will include sampling approach, data collection method, analytical approach, reported themes, contextual factors and authors’ interpretations. Data from mixed-methods studies will be extracted from both the quantitative and qualitative sections, and any point of integration between quantitative and qualitative findings will be recorded.

### Data analysis and evidence synthesis

Extracted data will be synthesised narratively to map the breadth of existing knowledge and to identify gaps requiring further research and targeted intervention. The narrative synthesis will follow the guidance outlined by Popay *et al*.^15^

All eligible study designs will be included. The primary outcomes of interest are reported mental health conditions and psychological distress, including but not limited to depression, anxiety, post-traumatic stress disorder, stress and psychosocial distress. Secondary outcomes include determinants of mental health, coping strategies, help-seeking pathways, access to services and findings from interventions.

Given the expected heterogeneity in populations, designs, measures and outcomes, a meta-analysis is not anticipated. Quantitative data will be reported using frequencies and qualitative findings will be analysed using thematic approaches to support the synthesis.^15^

If sufficient data are available, findings will be examined by subgroup, including sex, age group and population subgroups such as internally displaced people, women, adolescents or clinical populations. Intervention studies will be described and categorised separately, with attention to intervention type, delivery, acceptability and reported outcomes. Because meta-analysis is not anticipated, subgroup analysis will not involve pooled subgroup estimates. Instead, findings will be presented in tables and narrative synthesis, comparing the direction, range and context of findings across studies.

Quantitative and qualitative findings will be integrated using triangulation during interpretation to provide an overall account of mental health in Afghanistan, the factors influencing it and the extent to which services and interventions have been studied.^16^

### Critical appraisal of included studies

Study quality will be appraised using the Mixed Methods Appraisal Tool (MMAT), V.2018. The relevant MMAT criteria will be applied in accordance with the study design.^17^ Two reviewers will independently appraise the included studies, with disagreements resolved through discussion and, if necessary, by a third reviewer.^14^ Quality appraisal will not be used to exclude studies, but it will inform the interpretation of the findings.

### Patient and public involvement

Patients and the public were not involved in the design, conduct, reporting or dissemination plans of this research.

## Discussion

This systematic review will synthesise evidence on the mental health of people living in Afghanistan in the post-2021 context. The integration of findings from quantitative, qualitative and mixed-methods studies will provide a comprehensive understanding of the prevalence, determinants, coping strategies and service availability related to mental health in this population.

This review will provide evidence of the mental health effects of an evolving socio-political environment in Afghanistan since 2021. Decades of conflict, recent political change, economic instability and restrictions on fundamental rights—particularly for women—have likely exacerbated psychological distress across the population.

In addition to describing mental health outcomes, this review will examine the broader social and structural determinants influencing mental health, including poverty, displacement, gender-based restrictions and limited access to education and employment. These determinants are necessary to contextualise mental health within the lived realities of Afghan people, rather than viewing it solely through a clinical lens.

Evidence from conflict-affected and humanitarian settings in low- and middle-income countries shows a high burden of mental disorders, limited access to care and persistent barriers to scaling up mental health and psychosocial support.^18–20^ The Afghan evidence will therefore be interpreted with attention to both shared crisis-related patterns and features specific to Afghanistan after August 2021.

A further significant contribution of this review will be the exploration of coping strategies and help-seeking behaviours. In settings where formal mental health services are scarce, individuals often rely on informal support systems, religious practices and community networks. While these strategies may offer a certain degree of resilience, they may also be indicative of deficiencies in access to appropriate, culturally sensitive and evidence-based care. These findings can inform the development of targeted, feasible and context-appropriate interventions.

Of particular significance is the practical relevance of the findings of this review for policymakers, non-governmental organisations and humanitarian agencies. The identification of evidence-based needs and gaps is essential in facilitating the design of mental health programmes, community-based interventions and awareness initiatives. It may also inform the development of training workshops that are aimed at improving mental health literacy and reducing stigma among Afghan communities.

The heterogeneity of study designs, populations and outcome measures may limit the comparability of findings across studies. Moreover, the rapidly changing context in Afghanistan means that available data may not fully capture the current situation on the ground.

### Ethics and dissemination

This systematic review will not require ethical approval because it involves analysis of published literature only. Findings will be disseminated through peer-reviewed publication and conference presentations and may inform future research, clinical work and humanitarian and policy responses related to mental health in Afghanistan.

## Supplementary material

10.1136/bmjopen-2026-122310 online supplemental file 1

10.1136/bmjopen-2026-122310 online supplemental file 2
