# Supplementary material for: Mental health of people living in Afghanistan: protocol for a systematic review
Source: BMJ Open. 2026 Jul 31;16(7):e122310. doi: 10.1136/bmjopen-2026-122310 (PMC13436000; doi:10.1136/bmjopen-2026-122310)
Supplement: Supplementary file 1 [file bmjopen-2026-122310.reviewer_comments.pdf]

PEER REVIEW HISTORY

BMJ Open publishes all reviews undertaken for accepted manuscripts. Reviewers are asked to complete a checklist review form and are provided with free text boxes to elaborate on their assessment. These free text comments are reproduced below.

ARTICLE DETAILS

Title (Provisional)

Mental health of people living in Afghanistan: Protocol for a systematic review

Authors

Saeed, Heela; Kaya, Batu; Kuhn, Isla; Mughal, Faraz; Razai, Mohammad Sharif

---

VERSION 1 - REVIEW

Reviewer 1

Name Meyer, Thomas D.

Affiliation Univ Texas Hlth Sci Ctr Houston, Faillace Department of Psychiatry & Behav, Sci.

Date 09-Jun-2026

COI None

The authors propose a systematic review looking at the mental health of people living in Afghanistan. While Afghanistan is in a very specific and unique situation, I wonder if similar reviews have been published about other countries in crises, such as Iraq, Somalia, or Rwanda in the past, and whether it might sense to acknowledge them or to keep them in mind when drafting in the future the Discussion and putting the results potentially in an even larger context.

It is very positive that they plan to follow the standard PRISMA protocol and to register the protocol. When the authors list their strengths, one of them refers to the point that this will be the first review since 2021 which may or may not be a strength depending on the literature published since 2021. Perhaps it might be good to emphasize why that year might be special for obvious reasons as well.

When the authors describe in the Introduction a large scale, cross-sectional study (reference 6), perhaps add how depression and anxiety were assessed, self-rating, clinical interviews, structured interviews. This might be of interest to readers.

I was wondering if the thematic review that ‘included studies from 1976 to 2022’ actually consisted of publications (!) from 1976 to 2022 (i.e., the actual studies have been done

before but might just have been published in 2022)? I raise this with my former comment in mind about the year 2021 in the ‘strength’ section.

Looking at inclusion and exclusion criteria, it would be good to provide an even stronger rational why only to consider studies published from 2021 onwards, because one could make an argument that looking directly at trends over time would be a good idea and very informative as well.

Please specify how non-English articles will be translated.

It would be interesting why the authors chose to word the following as an exclusion instead of an inclusion criterion: ‘Study does not focus on people living inside Afghanistan’ (instead of ‘Study focuses on people living inside Afghanistan’. I bring this up because I was almost tempted to ask what the difference is between the two first exclusion criteria. At first, they seemed to say the same till I reflected on it longer. Therefore, rewording it as an inclusion criterion might be clarifying.

About the screening of records and assessment of study quality/bias, it is stated that two independent reviewers will decide. Consider perhaps for all discrepant decisions to involve a third reviewer instead of a discussion. Rather use the discussion if the third reviewer finds it difficult to decide and needs to hear the rational from the other two. Are you planning to disclose the reasons for exclusions in line with the inclusion/exclusion criteria? If so, please state that explicitly.

I appreciate the idea of a standardized data extraction form. I just wonder how easy that will be for including both quantitative and qualitative studies to do both (or their mix) justice? Perhaps elaborate on that a little, how this will look like.

The authors write ‘when sufficient data are available.’…. I was wondering a) if it should be worded ‘if sufficient data are available...’ and b) how would these findings be examined by subgroups when the authors themselves do not expect sufficient data homogeneity to do a meta-analysis? Please elaborate on that.

Please check if you could provide for references 12 and 13 more information about where to locate and retrieve the information.

Appendix PRISMA 2020 Checklist: I leave it to the Editors, but I am not sure if the PRISMA checklist is needed as part of the published protocol.

Last but not least, I was also wondering if the authors plan to update the searches to June/July 2026 to cover the current literature as much as possible?

VERSION 1 - AUTHOR RESPONSE

We thank the editor and reviewer for their careful and constructive comments. We believe the suggestions and feedback have improved the paper. Please find below our response to the points raised.

COMMENT	RESPONSE	EDITS IN MANUSCRIPT with track changes
**Reviewer 1**	**Response**	**Edits in manuscript With track changes**
The authors propose a systematic review looking at the mental health of people living in Afghanistan. While Afghanistan is in a very specific and unique situation, I wonder if similar reviews have been published about other countries in crises, such as Iraq, Somalia, or Rwanda in the past, and whether it might sense to acknowledge them or to keep them in mind when drafting in the future the Discussion and putting the results potentially in an even larger context.	Thank you. We agree that the review should be placed in the wider literature on mental health in humanitarian and post-conflict settings. We have added text to the *Discussion* noting relevant evidence from conflicted affected low and middle income settings and that the Afghan evidence will be interpreted in relation to both shared crisis-related patterns and features specific to Afghanistan after August 2021.	Page 12
It is very positive that they plan to follow the standard PRISMA protocol and to register the protocol. When the authors list their strengths, one of them refers to the point that this will be the first review since 2021 which may or may not be a strength depending on the literature published since 2021.	We have also removed the claim about being the first review from the Strengths and limitations section as suggested.	Page 4
Perhaps it might be good to emphasize why that year might be special for obvious reasons as well.	As suggested, we have strengthened the rationale for the 2021 cut-off in the *Eligibility criteria* section.	Page 6
When the authors describe in the Introduction a large scale, cross-sectional study (reference 6), perhaps add how depression and anxiety were assessed, self-rating, clinical interviews, structured interviews. This might be of interest to readers.	Thank you. We have revised this sentence to state that the study used the 21-item Depression, Anxiety and Stress Scale (DASS-21). We have also described the findings as symptoms rather than diagnoses, to avoid overstating what the instrument can establish.	Page 5
I was wondering if the thematic review that 'included studies from 1976 to 2022' actually consisted of publications (!) from 1976 to 2022 (i.e., the actual studies have been done before but might just have been published in	Thank you. We have clarified that the earlier thematic review was published in 2023 through searches conducted in 2022. This also helps distinguish the earlier	Page 5

2022)? I raise this with my former comment in mind about the year 2021 in the 'strength' section.	four-decade synthesis from the current post-2021 review.	
Looking at inclusion and exclusion criteria, it would be good to provide an even stronger rational why only to consider studies published from 2021 onwards, because one could make an argument that looking directly at trends over time would be a good idea and very informative as well.	Thank you. We have expanded the rationale in the introduction for including studies published from 2021 onwards. The revised text explains that August 2021 marked a major change in governance, restrictions on education, employment and mobility for women, reductions in international aid, and disruption to health and social systems.	Page 6
Please specify how non-English articles will be translated.	Thank you for this helpful suggestion. We have revised the *Eligibility criteria* section to specify how non-English articles will be handled. The revised text explains that translation will be conducted by reviewers with relevant language expertise and professional translations services where feasible. Machine translation may be used where appropriate.	Page 8
It would be interesting why the authors chose to word the following as an exclusion instead of an inclusion criterion: 'Study does not focus on people living inside Afghanistan' (instead of 'Study focuses on people living inside Afghanistan'. I bring this up because I was almost tempted to ask what the difference is between the two first exclusion criteria. At first, they seemed to say the same till I reflected on it longer. Therefore, rewording it as an inclusion criterion might be clarifying.	Thank you. We agree that the wording was potentially confusing. We have revised this section as suggested.	Pages 7 and 8
About the screening of records and assessment of study quality/bias, it is stated that two independent reviewers will decide. Consider perhaps for all discrepant decisions to involve a third reviewer instead of a discussion.	Thank you. We agree that the process for resolving disagreements should be stated more clearly. We have revised the manuscript to specify that screening and quality appraisal	Page 10

Rather use the discussion if the third reviewer finds it difficult to decide and needs to hear the rational from the other two.	will be undertaken independently by two reviewers. Disagreements will first be resolved through discussion, as this allows errors or misunderstandings to be corrected efficiently. If consensus cannot be reached, a third reviewer will adjudicate. We consider this approach consistent with standard systematic review practice, including Cochrane guidance, which recognises discussion as an appropriate first step and arbitration by another reviewer where disagreements reflect differences in interpretation.	
Are you planning to disclose the reasons for exclusions in line with the inclusion/exclusion criteria? If so, please state that explicitly.	As suggested, we have also stated that reasons for exclusion at full-text stage will be recorded and reported in the PRISMA flow diagram.	Page 10
I appreciate the idea of a standardized data extraction form. I just wonder how easy that will be for including both quantitative and qualitative studies to do both (or their mix) justice? Perhaps elaborate on that a little, how this will look like.	Thank you. We have expanded the data extraction section. The revised text explains that the form will include a core section for all studies and design-specific sections for quantitative, qualitative, and mixed-methods studies. This will allow the review to capture prevalence estimates, measurement tools, effect estimates, qualitative themes, contextual factors, and points of integration in mixed-methods studies.	Page 10
The authors write ‘when sufficient data are available.’…. I was wondering a) if it should be worded ‘if sufficient data are available...’ and b) how would these findings be examined by subgroups when the authors themselves do not expect sufficient data	Thank you. We have changed “where sufficient data are available” to “if sufficient data are available” and clarified that subgroup examination will be descriptive. Because meta-analysis is not anticipated, subgroup findings will be	Page 11

homogeneity to do a meta-analysis? Please elaborate on that.	presented through tables and narrative synthesis, comparing the direction, range, and context of findings across studies.	
Please check if you could provide for references 12 and 13 more information about where to locate and retrieve the information.	Thank you. We have replaced the incomplete entries for references 12 and 13 with full bibliographic details.	These references are numbers 8 and 15 in the revised manuscript
Appendix PRISMA 2020 Checklist: I leave it to the Editors, but I am not sure if the PRISMA checklist is needed as part of the published protocol.	Thank you. We have clarified the reporting statement so that the protocol follows PRISMA-P and the completed PRISMA-P checklist is provided as supplementary file. The completed review will be reported in accordance with PRISMA 2020. We have retained the checklist because the editor specifically requested it.	Supplemental file 1.
Last but not least, I was also wondering if the authors plan to update the searches to June/July 2026 to cover the current literature as much as possible?	Thank you. We have added that the searches will be updated before final synthesis depending on the timing of manuscript finalisation.	Abstract and Page 8

VERSION 2 - REVIEW

Reviewer 1

Name Meyer, Thomas D.

Affiliation Univ Texas Hlth Sci Ctr Houston, Faillace Department of Psychiatry & Behav, Sci.

Date 10-Jul-2026

COI

The authors have sufficiently addressed my comments and suggestions. I have no further comments, and just wish them the best for doing the actual work and publishing it.
