# Supplementary material for: Mental health of people living in Afghanistan: protocol for a systematic review
Source: BMJ Open. 2026 Jul 31;16(7):e122310. doi: 10.1136/bmjopen-2026-122310 (PMC13436000; doi:10.1136/bmjopen-2026-122310)
Supplement: Supplementary file 3 [file bmjopen-16-7-s002.docx]

Mental health of people living in Afghanistan: Protocol for a systematic review

| Medline via Ovid | 552 |
| --- | --- |
| PsycINFO via ebscohost | 443 |
| WHO Global Index Medicus | 3 |
| Total | 998 |
| Total deduplicated | 761 |

Publication date limit: 2021 to current

Searches run 4^th^ Feb 2026

# Medline

Ovid MEDLINE(R) and Epub Ahead of Print, In-Process, In-Data-Review & Other Non-Indexed Citations, Daily and Versions <1946 to February 03, 2026>

1	(afghan* or kabul or herat or kandahar or kunduz or mazar-i-sharif or jalalabad or sistan or helmand or hindu kush or pamir).ti,ab,kw,kf.	10324

2	exp Afghanistan/	4121

3	1 or 2	11080

4	(mental health or mental* ill*).ti,ab,kw,kf.	336359

5	exp Mental Health/	76836

6	exp Mental Disorders/	1588027

7	(anxiety or depression or psychosis or psychotic or mental wellbeing or mental trauma or ptsd or post-traumatic stress or posttraumatic stress).ti,ab,kw,kf.	781530

8	exp Stress Disorders, Post-Traumatic/	47420

9	exp Anxiety/ or exp Anxiety Disorders/	214027

10	exp Depression/	178265

11	exp Psychotic Disorders/	62395

12	or/4-11	2246153

13	3 and 12	2444

14	limit 13 to yr="2021 -Current"	552

# PsycINFO

EBSCO Export

| **S#** | S8 |
| --- | --- |
| **Query (user-entered)** | S5 AND S1 |
| **Query (expanded/display term)** | ((DE "Mental Disorders" OR DE "Affective Disorders" OR DE "Anxiety Disorders" OR DE "Behavior Disorders" OR DE "Bipolar Disorder" OR DE "Borderline States" OR DE "Chronic Mental Illness" OR DE "Dissociative Disorders" OR DE "Eating Disorders" OR DE "Gender Dysphoria" OR DE "Mental Disorders due to General Medical Conditions" OR DE "Neurocognitive Disorders" OR DE "Neurodevelopmental Disorders" OR DE "Neurosis" OR DE "Obsessive Compulsive Disorder" OR DE "Paraphilic Disorders" OR DE "Personality Disorders" OR DE "Psychosis" OR DE "Serious Mental Illness" OR DE "Sleep Wake Disorders" OR DE "Somatoform Disorders" OR DE "Stress and Trauma Related Disorders" OR DE "Substance Related and Addictive Disorders" OR DE "Thought Disorders" OR DE "Disruptive Mood Dysregulation Disorder" OR DE "Major Depression" OR DE "Anaclitic Depression" OR DE "Endogenous Depression" OR DE "Late Life Depression" OR DE "Postpartum Depression" OR DE "Reactive Depression" OR DE "Recurrent Depression" OR DE "Seasonal Affective Disorder" OR DE "Treatment Resistant Depression" OR DE "Persistent Depressive Disorder" OR DE "Dysthymic Disorder" OR DE "Premenstrual Dysphoric Disorder" OR DE "Castration Anxiety" OR DE "Generalized Anxiety Disorder" OR DE "Panic Attack" OR DE "Panic Disorder" OR DE "Phobias" OR DE "Acrophobia" OR DE "Agoraphobia" OR DE "Claustrophobia" OR DE "Fear of Failure" OR DE "Ophidiophobia" OR DE "School Phobia" OR DE "Social Phobia" OR DE "Xenophobia" OR DE "Selective Mutism" OR DE "Separation Anxiety Disorder" OR DE "Social Anxiety Disorder" OR DE "Conduct Disorder" OR DE "Disruptive Behavior Disorders" OR DE "Oppositional Defiant Disorder" OR DE "Emotional and Behavioral Disorders" OR DE "Emotional Disturbances" OR DE "Impulse Control Disorders" OR DE "Explosive Disorder" OR DE "Kleptomania" OR DE "Pyromania" OR DE "Self-Destructive Behavior" OR DE "Nonsuicidal Self-Injury" OR DE "Head Banging" OR DE "Self-Inflicted Wounds" OR DE "Self-Poisoning" OR DE "Suicidal Behavior" OR DE "Attempted Suicide" OR DE "Suicidal Ideation" OR DE "Suicidality" OR DE "Suicide" OR DE "Military Suicide" OR DE "Youth Suicide" OR DE "Bipolar I Disorder" OR DE "Bipolar II Disorder" OR DE "Cyclothymic Disorder" OR DE "Mania" OR DE "Hypomania" OR DE "Chronic Psychosis" OR DE "Depersonalization" OR DE "Depersonalization/Derealization Disorder" OR DE "Dissociative Amnesia" OR DE "Dissociative Fugue" OR DE "Dissociative Identity Disorder" OR DE "Anorexia Nervosa" OR DE "Avoidant/Restrictive Food Intake Disorder" OR DE "Binge Eating Disorder" OR DE "Bulimia" OR DE "Feeding Disorders" OR DE "Hyperphagia" OR DE "Kleine Levin Syndrome" OR DE "Orthorexia" OR DE "Pica" OR DE "Purging (Eating Disorders)" OR DE "Rumination (Eating)" OR DE "Delirium" OR DE "Dementia" OR DE "AIDS Dementia Complex" OR DE "Alzheimer's Disease" OR DE "Dementia with Lewy Bodies" OR DE "Frontotemporal Lobar Degeneration" OR DE "Frontotemporal Dementia" OR DE "Picks Disease" OR DE "Huntingtons Disease" OR DE "Presenile Dementia" OR DE "Pseudodementia" OR DE "Senile Dementia" OR DE "Vascular Dementia" OR DE "Memory Disorders" OR DE "Amnesia" OR DE "Anterograde Amnesia" OR DE "Global Amnesia" OR DE "Retrograde Amnesia" OR DE "Mild Cognitive Impairment" OR DE "Attention Deficit Disorder" OR DE "Attention Deficit Disorder with Hyperactivity" OR DE "Autism Spectrum Disorders" OR DE "Autistic Traits" OR DE "Communication Disorders" OR DE "Language Disorders" OR DE "Agnosia" OR DE "Anosognosia" OR DE "Prosopagnosia" OR DE "Agraphia" OR DE "Dyscalculia" OR DE "Dyslexia" OR DE "Alexia" OR DE "Echolalia" OR DE "Mutism" OR DE "Specific Language Impairment" OR DE "Speech Disorders" OR DE "Aphasia" OR DE "Articulation Disorders" OR DE "Dysarthria" OR DE "Dysphasia" OR DE "Dysphonia" OR DE "Speech Sound Disorder" OR DE "Stuttering" OR DE "Social Communication Disorder" OR DE "Intellectual Development Disorder" OR DE "Crying Cat Syndrome" OR DE "Down's Syndrome" OR DE "Tay Sachs Disease" OR DE "Learning Disabilities" OR DE "Learning Disorders" OR DE "Reading Disabilities" OR DE "Motor Disorders" OR DE "Developmental Coordination Disorder" OR DE "Stereotypic Movement Disorder" OR DE "Tic Disorders" OR DE "Tourette Syndrome" OR DE "Childhood Neurosis" OR DE "Experimental Neurosis" OR DE "Traumatic Neurosis" OR DE "Body Dysmorphic Disorder" OR DE "Muscle Dysmorphia" OR DE "Excoriation Disorder" OR DE "Hoarding Disorder" OR DE "Koro" OR DE "Trichotillomania" OR DE "Exhibitionism" OR DE "Fetishism" OR DE "Pedophilia" OR DE "Sexual Masochism" OR DE "Sexual Sadism" OR DE "Voyeurism" OR DE "Antisocial Personality Disorder" OR DE "Avoidant Personality Disorder" OR DE "Borderline Personality Disorder" OR DE "Dependent Personality Disorder" OR DE "Histrionic Personality Disorder" OR DE "Hysteria" OR DE "Mass Hysteria" OR DE "Narcissistic Personality Disorder" OR DE "Obsessive Compulsive Personality Disorder" OR DE "Paranoid Personality Disorder" OR DE "Passive Aggressive Personality Disorder" OR DE "Sadomasochistic Personality" OR DE "Masochistic Personality" OR DE "Schizoid Personality Disorder" OR DE "Schizotypal Personality Disorder" OR DE "Affective Psychosis" OR DE "Brief Psychotic Disorder" OR DE "Postpartum Psychosis" OR DE "Capgras Syndrome" OR DE "Childhood Onset Psychosis" OR DE "Childhood Onset Schizophrenia" OR DE "Delusional Disorder" OR DE "Shared Paranoid Disorder" OR DE "Experimental Psychosis" OR DE "Hallucinosis" OR DE "Alcohol Withdrawal Syndrome" OR DE "Korsakoff Syndrome" OR DE "Wernicke Encephalopathy" OR DE "Paranoid Psychosis" OR DE "Reactive Psychosis" OR DE "Schizophrenia" OR DE "Acute Schizophrenia" OR DE "Catatonic Schizophrenia" OR DE "Paranoid Schizophrenia" OR DE "Process Schizophrenia" OR DE "Schizoaffective Disorder" OR DE "Schizophrenia (Disorganized Type)" OR DE "Schizophreniform Disorder" OR DE "Undifferentiated Schizophrenia" OR DE "Substance Induced Psychotic Disorders" OR DE "Alcohol Induced Psychotic Disorders" OR DE "Hypersomnia" OR DE "Insomnia" OR DE "Narcolepsy" OR DE "Parasomnias" OR DE "Bruxism" OR DE "REM Sleep Behavior Disorder" OR DE "Restless Leg Syndrome" OR DE "Sleep Arousal Disorders" OR DE "Sleep Terrors" OR DE "Sleepwalking" OR DE "Sleep Apnea" OR DE "Sleep-Related Hypoventilation" OR DE "Factitious Disorders" OR DE "Munchausen Syndrome" OR DE "Munchausen Syndrome by Proxy" OR DE "Functional Neurological Disorder" OR DE "Functional Paralysis" OR DE "Functional Visual Symptoms" OR DE "Pseudocyesis" OR DE "Illness Anxiety Disorder" OR DE "Neurasthenia" OR DE "Somatization Disorder" OR DE "Somatoform Pain Disorder" OR DE "Acute Stress Disorder" OR DE "Adjustment Disorders" OR DE "Attachment Disorders" OR DE "Disinhibited Social Engagement Disorder" OR DE "Posttraumatic Stress Disorder" OR DE "Complex PTSD" OR DE "DESNOS" OR DE "Prolonged Grief Disorder" OR DE "Addiction" OR DE "Drug Addiction" OR DE "Drug Withdrawal" OR DE "Alcohol Withdrawal" OR DE "Nicotine Withdrawal" OR DE "Opioid Withdrawal" OR DE "Nonsubstance Related Addictions" OR DE "Compulsive Shopping" OR DE "Exercise Dependence" OR DE "Food Addiction" OR DE "Gambling Disorder" OR DE "Internet Addiction" OR DE "Internet Gaming Disorder" OR DE "Sexual Addiction" OR DE "Workaholism" OR DE "Substance Use Disorder" OR DE "Alcohol Use Disorder" OR DE "Alcohol Abuse" OR DE "Alcoholism" OR DE "Binge Drinking" OR DE "Cannabis Use Disorder" OR DE "Drug Abuse" OR DE "Polydrug Abuse" OR DE "Drug Dependency" OR DE "Inhalant Abuse" OR DE "Glue Sniffing" OR DE "Opioid Use Disorder" OR DE "Heroin Use Disorder" OR DE "Morphine Dependence" OR DE "Tobacco Use Disorder" OR DE "Confabulation" OR DE "Delusions" OR DE "Fantasies (Thought Disturbances)" OR DE "Fixation (Psychological)" OR DE "Fragmentation (Schizophrenia)" OR DE "Intrusive Thoughts" OR DE "Judgment Disturbances" OR DE "Magical Thinking" OR DE "Obsessions" OR DE "Paranoia" OR DE "Perseveration" OR DE "Rumination (Cognitive Process)") OR (XB (anxiety or depression or psychosis or psychotic or mental wellbeing or mental trauma or ptsd or post-traumatic stress or posttraumatic stress)) OR (XB (mental health or mental* ill*))) AND (XB (afghan* or kabul or herat or kandahar or kunduz or mazar-i-sharif or jalalabad or sistan or helmand or hindu kush or pamir)) |
| **Search run Date and Time** | 2026-02-04T14:06:05.242Z |
| **Results (count)** | 443 |
| **Search Mode** | Proximity |
| **Expander(s)** | Apply equivalent subjects |
| **Filter(s)** |  |
| **Publication Date (start date)** | 2021-01-01 |
| **Publication Date (end date)** | 2027-02-28 |
| **Interface** | EBSCOhost Research Databases |
| **Database(s)** | APA PsycInfo |

# WHO Global Index Medicus

Title, abstract, subject: (afghan* or kabul or herat or kandahar or kunduz or mazar-i-sharif or jalalabad or sistan or helmand or hindu kush or pamir) and (mental health or mental* ill* or anxiety or depression or psychosis or psychotic or mental wellbeing or mental trauma or ptsd or post-traumatic stress or posttraumatic stress)
